# Glucose Kinetics in the Collagen-Induced Arthritis Model: An All-in-One Model to Assess Both Efficacy and Metabolic Side Effects of Glucocorticoids

**DOI:** 10.1371/journal.pone.0098684

**Published:** 2014-09-02

**Authors:** Erik J. M. Toonen, Anke J. Laskewitz, Theo H. van Dijk, Aycha Bleeker, Aldo Grefhorst, Annelies E. Schouten, Ellen A. J. Bastiaanssen, Dov B. Ballak, Marije I. Koenders, Cindy van Doorn, Monique A. J. van der Vleuten, Marie-Jose C. van Lierop, Albert K. Groen, Wim H. A. Dokter

**Affiliations:** 1 Department of Internal Medicine, Radboud University Medical Centre, Nijmegen, The Netherlands; 2 Department of Pediatrics, University of Groningen, University Medical Center Groningen, Groningen, The Netherlands; 3 Department of Laboratory Medicine, University of Groningen, University Medical Center Groningen, Groningen, The Netherlands; 4 Department of Internal Medicine, Erasmus MC, University Medical Center Rotterdam, Rotterdam, The Netherlands; 5 Department of Immune Therapeutics, MSD Research Laboratories, Oss, The Netherlands; 6 Department of Experimental Rheumatology, Radboud Institute for Molecular Life Sciences (RIMLS), Radboud University Medical Centre, Nijmegen, The Netherlands; Shanghai Jiaotong University School of Medicine, China

## Abstract

Prednisolone and other glucocorticoids (GCs) are potent anti-inflammatory drugs, but chronic use is hampered by metabolic side effects. Therefore, there is an urgent medical need for improved GCs that are as effective as classical GCs but have a better safety profile. A well-established model to assess anti-inflammatory efficacy is the chronic collagen-induced arthritis (CIA) model in mice, a model with features resembling rheumatoid arthritis. Models to quantify undesired effects of glucocorticoids on glucose kinetics are less well-established. Recently, we have described a model to quantify basal blood glucose kinetics using stably-labeled glucose. In the present study, we have integrated this blood glucose kinetic model in the CIA model to enable quantification of both efficacy and adverse effects in one animal model. Arthritis scores were decreased after treatment with prednisolone, confirming the anti-inflammatory properties of GCs. Both inflammation and prednisolone induced insulin resistance as insulin secretion was strongly increased whereas blood glucose concentrations and hepatic glucose production were only slightly decreased. This insulin resistance did not directly resulted in hyperglycemia, indicating a highly adaptive compensatory mechanism in these mice. In conclusion, this ‘all-in-one’ model allows for studying effects of (novel) GC compounds on the development of arthritis and glucose kinetics in a single animal. This integrative model provides a valuable tool for investigating (drug-induced) metabolic dysregulation in an inflammatory setting.

## Introduction

Prednisolone and other glucocorticoids (GCs) are very potent immunosuppressive and anti-inflammatory compounds that are among the top 10 most prescribed drugs [1]. Exogenous GCs are used in daily clinical practice to treat (chronic) inflammatory, autoimmune and allergic disorders, to attenuate organ rejection after transplantation, to treat brain edema, shock and various blood cancers [2]. Most of the effects of GCs are mediated through binding of GCs to the glucocorticoid receptor (GR), which is found in almost all human tissues.

Although very effective in reducing inflammation, prolonged treatment at medium or high dose of GCs is hampered by a wide range of metabolic side effects such as derangements of glucose metabolism, induction of insulin-resistance, beta-cell dysfunction, hyperlipidemia, fat redistribution and central obesity that are all strongly associated with elevated risk for cardiovascular disease and type 2 Diabetes mellitus in humans [3]–[5]. The mechanisms of action underlying these metabolic side effects are largely unknown.

Seen this wide range of GC-induced side effects, there is a high medical need for improved anti-inflammatory drugs that are as effective as classical GCs but show less metabolic side effects. This type of experimental compounds are often referred to as selective GR modulators (SGRMs) [4]. Given the complexity of glucocorticoid actions, the use of animal models is required to investigate these mechanisms and several animal models have been applied to study the efficacy and/or metabolic side effects of new pharmaceutical compounds *in vivo* [6], [7]. Preclinical efficacy of experimental GCs is often measured in collagen-induced arthritis (CIA) mice, a chronic inflammatory model with features resembling rheumatoid arthritis (RA) [8], [9]. Arthritis can be induced in susceptible strains of mice by immunization with type II collagen, the major component of articular cartilage, and has histopathologic and serological features in common with RA [10], [11]. Regarding *in vivo* investigation of (compound-induced) metabolic dysregulation, such as insulin resistance, a number of methods have been developed to measure glucose and insulin kinetics, of which the hyperinsulinemic euglycemic clamp (HIEC) is considered as the ‘gold standard’. With respect to mice studies, the HIEC is difficult to use for longitudinal studies, since it can only be performed once in a single animal [12]. In addition, arthritic mice in the CIA model are severely ill, with highly inflamed joints and therefore these mice can not be subjected to the invasive HIEC protocol. To overcome these drawbacks, a new method was developed in which stably-labeled glucose (D-[6,6-^2^H_2_]-glucose) was used in combination with a single-pool, first order kinetic model to determine blood glucose kinetics [13]. This model is especially of interest for longitudinal studies, due to the ability of repeated measurements.

Currently, an animal model to study both efficacy and glucocorticoid-induced metabolic side effects in a chronic inflammatory setting is not available. Therefore, the aim of the present study is to develop a chronic inflammatory model which can be used to investigate both efficacy *and* metabolic safety of GCs in the same animal at the same time. To do so, we have integrated the newly developed blood glucose kinetics model into the CIA mice model.

To validate this model and to investigate the mechanisms involved in GC-induced metabolic dysfunction in a chronic inflammatory setting, we performed two consecutive CIA experiments. First, we assessed the effects of several doses of prednisolone (0, 1.5, 10 and 30 mg/kg/day) on efficacy and metabolic safety after 0, 7 and 21 days of treatment. Next, in order to distinguish between direct effects of prednisolone and indirect effects on glucose kinetics resulting from its anti-inflammatory efficacy, we compared effects of prednisolone to those of ORG 37663. This compound is a steroid which has previously been described to be effective for the treatment of arthritis in CIA mice, but mediates its effect through the estrogen receptor alpha (ERα) instead of the GR (6). Comparing the results from the prednisolone-treated subgroup to the subgroup treated with the anti-inflammatory compound ORG 37663 allowed us to distinguish between direct effects of prednisolone on glucose and insulin kinetics and indirect effects induced by the anti-inflammatory properties of prednisolone.

The results from this study reveal that it was possible to measure both efficacy and metabolic side effects of prednisolone in one experimental set-up. Arthritis scores were dose-dependently decreased in arthritic mice after treatment with both prednisolone and ORG 37663. Concerning effects on glucose metabolism, there was a decrease in insulin sensitivity in both arthritic and non-arthritic control mice after treatment with prednisolone for three weeks, while ORG 37663 unexpectedly increased insulin sensitivity in both groups. These prednisolone-mediated effects on insulin sensitivity were mainly reflected by an increased insulin secretion.

Overall, the integration of these two models provides a valuable tool for studying both the efficacy *and* metabolic side-effects of experimental glucocorticoids at the same time in one model.

## Methods

### Animals

All experiments were approved by the Animal Welfare Committee of MSD Oss, The Netherlands. Male DBA1/J mice were obtained from Bomholtgard (Ry, Denmark). Animals were housed and maintained at 23°C with *ad libitum* access to water and food in a 12 hour-12 hour light-dark cycle (lights on 6 am-6 pm).

### Therapeutic murine collagen-induced arthritis

The murine CIA model was performed as previously described [14]. In brief, DBA1/J mice were immunized at the base of the tail at the age of eight weeks with 200 µg bovine type II collagen in complete Freud's adjuvant enriched with 2 mg/ml *M. tuberculosis* (H37Ra). Three weeks after immunization the animals were boosted with an intraperitoneal injection of 200 µg collagen type II, dissolved in saline. Earlier experiments have taught us that, after immunization, mice will drop-out due to an early aggressive arthritis or fail to develop arthritis. About 60–70% of the mice develop the necessary arthritis. Therefore, the total amount of mice that are immunized and boosted for the experiment is 40% above the actual number of mice needed for the experiment. The clinical severity of arthritis (arthritis score) was graded per inflamed digit (a scale of 0 to 2 for each paw). Mice were scored on alternative days, resulting in mean scores with an overall maximum of 8 per animal. To assess the effects of treatment, the area under curve (AUC) of the mean arthritis of each animal with baseline correction (subtracting baseline AUC of arthritis score on day 0) was used. After disease onset, animals with an arthritis score ranging from 2 to 4 were divided into separate groups of 12 mice so that the mean arthritis score of all experimental groups was comparable at the start of the treatment (day 0). Mice were considered to have arthritis when significant changes in redness and/or swelling were noted in the digits or in other parts of the paws.

### Treatment and experimental regimes

For the first experiment, a total of 40 arthritic animals were divided into an experimental and a control group of 20 mice each. Both groups were orally treated once a day for 21 days with placebo (0.5% gelatin and 5% mannitol in water) and arthritis development was monitored. In the experimental group (n = 20) mice were subjected to a blood glucose kinetics test three times (on day 0, 7 and 21) while mice in the control group were not subjected to the blood glucose kinetics tests.

For the second experiment, arthritic animals were treated orally once a day for 21 days with either 1.5, 10 or 30 mg/kg prednisolone in vehicle (0.5% gelatin and 5% mannitol in water) or vehicle alone as placebo. In total, 48 arthritic mice (12 per treatment group) were included. For non-arthritic control groups, mice were mock immunized with saline at the base of the tail at the age of eight weeks and three weeks later mock boosted with saline. Mice were further treated according the same experimental protocol and handled exactly the same as the mice that developed arthritis. In this study, these mice are referred to as non-arthritic control mice.

For the third experiment, arthritic mice were treated orally once a day for 21 days either with prednisolone (10 mg/kg) or ORG 37663 (12 mg/kg) in vehicle (0.5% gelatin and 5% mannitol in water) or vehicle alone as placebo. The dose of 12 mg/kg/day for ORG 37663 was selected because this dose is comparable to a dose of 10 mg/kg/day prednisolone regarding efficacy [6]. In total, 36 arthritic mice (12 per treatment group) and 36 non-arthritic control mice, like described above, were included in this consecutive experiment.

All experimental treatments were conducted in a blinded fashion. At the end of the experiment, serum samples and hind knees and paws were obtained. Hind knees and paws were evaluated using X-ray analysis to assess bone destruction [15]. X-ray photographs were examined with a Faxitron X-ray MX-20 (0.02 mm resolution) and bone destruction was scored on a scale from 0 to 5 ranging from no damage to complete destruction [16].

### Blood glucose kinetics

The fasted blood glucose kinetic test was performed three times; at day 0 (before treatment, but after disease onset) and after 7 and 21 days of treatment. For each experiment, mice were fasted for 9 hours overnight (11.00 pm–8.00 am), body weights were measured and mice were injected intraperitoneally with a small volume of 2.0 mg D-[6,6-^2^H_2_]glucose in 0.20 ml by intraperitoneal injection (∼450 µmol/kg BW) which did not cause changes in blood glucose and plasma insulin concentrations. Before and at 10, 20, 30, 40, 50, 60, 75 and 90 minutes after D-[6,6-^2^H_2_]-glucose administration, blood glucose concentrations were measured in a blood drop collected by tail tip bleeding using a glucocard X-meter (A. Menarini Diagnostics; Valkenswaard; The Netherlands). At the same time points, a small blood spot was taken on filter paper (FT-2-460-210297, Sartorius, Goettingen, Germany) and stored at room temperature until further analysis of D-[6,6-^2^H_2_]-glucose label distribution. After the last test (day 21) a blood sample (15 µl) was taken by tail tip bleeding for C-peptide measurements.

### Measurements of mass isotopomer distribution by GC-MS

Whole body glucose turnover and clearance were calculated by kinetic analysis from the wash-out of injected D-[6,6-^2^H_2_]-glucose from circulation for which blood spots on filter paper were taken. Extraction of glucose from filter paper, derivatization of the extracted compounds and gas chromatography-mass spectrometry (GC-MS) measurements of blood glucose was performed as previously described by Van Dijk and colleagues [17]. In short, a disk was punched out of the blood spots, glucose was extracted from the disk by incubation in ethanol/water (10∶1 v/v) and glucose was derivatized to its pentaacetate-ester. Samples were analyzed by GC-MS with positive ion chemical ionization with ammonia. The fractional isotopomer distribution measured by GC-MS (m_0_-m_6_) was corrected for the fractional distribution due to natural abundance of ^13^C by multiple linear regression as described by Lee *et al*. [18] to obtain the excess fractional distribution of mass isotopomers (M_0_-M_6_) due to dilution of infused labeled compounds.

### Calculation of blood glucose kinetics

For calculating the blood glucose kinetics parameters EGP (Endogenous Glucose Production) and MCR (Metabolic Clearance Rate), a single-pool, first-order kinetic model was assumed [19]–[21]. The excess fractional distribution of mass isotopomers (M_2_) was used to calculate the first order absorption process in an one-compartment model using SAAM-II software (version 1.2.1; SAAM Institute, University of Washington, Seattle, WA, USA) [13]. The formulas used to calculate the concentration *vs*. time curves and the kinetic parameters are outlined in Table 1.

**Table 1:** The formulas used to calculate the concentration *vs*. time curves and the kinetic parameters in a first order absorption process in an one-compartment model.

Eq. 1	Tracer Concentration at time point t	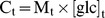
Eq. 2	Single-pool first-order kinetics	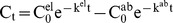
Eq. 3	Bioavailability	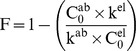
Eq. 4	Area under the curve	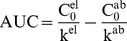
Eq. 5	Metabolic clearance rate	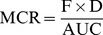
Eq. 6	Apparent volume of distribution	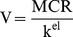
Eq. 7	Turnover rate	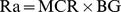
Eq. 8	Pool size	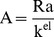

C_t_, D-[6,6-^2^H]-glucose concentration at time point t; M_t_, fractional contribution of D-[6,6-^2^H]-glucose at time point t; [glc]_t_, blood glucose concentration at time point t. C(0)^ab^, initial concentration of D-[6,6-^2^H]-glucose determined by extrapolation of the absorption period; C(0)^el^, initial concentration of D-[6,6-^2^H]-glucose determined by extrapolation of the elimination period; k^ab^, absorption rate constant; k^el^, absorption rate constant. D, dose D-[6,6-^2^H]-glucose administrated; BG, average blood glucose concentration during the test; Ra = EGP.

### C-peptide measurements

In this study C-peptide concentrations as a marker for insulin production, since the half-life of C-peptide is longer than that of insulin. Thereby, by measuring C-peptide we can focus on de novo synthesis of insulin and not just circulating insulin that might also be released/taken up by cells. Peripheral blood samples obtained after each experiment were centrifuged and the retrieved serum samples were stored at −20°C until analyzed. C-peptide concentrations were measured using a commercially available ELISA kit (mouse C-peptide ELISA; Alpco immunoassays, Salem, NH, USA).

### Calculation of HOMA-IR

The Homeostasis Model Assessment for Insulin Resistance (HOMA-IR) combines fasting glucose and insulin concentrations to calculate insulin resistance [22]. Besides using insulin, it is also possible to calculate the HOMA-IR using C-peptide concentrations [23]. The HOMA-IR expresses changes in insulin sensitivity and β-cell function relative to a population in which these parameters are considered to be normal. However, although the HOMA-IR has been used for mice studies previously, it was developed based upon human values. Therefore we adjusted the HOMA-IR for mice in which the healthy mice, treated with placebo, had an insulin resistance (IR) of 1 (no insulin resistance). This group is used as the reference group. The reference value can be calculated from median blood glucose concentration (6.6 mM) during the blood glucose kinetics protocol and the mean plasma C-peptide concentration (221 pM) at the end of the test. For this study, the value was 1459. In this manuscript, the HOMA-IR was therefore calculated as:



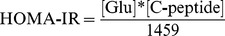



In which [Glu] is the blood glucose concentration in mM and [C-peptide] is the C-peptide plasma concentration in pM. An HOMA-IR >1 indicates induction of insulin resistance and an HOMA-IR <1 indicates improved insulin sensitivity.

### Insulin tolerance test

For the insulin tolerance test, mice were fasted for 4 hours before i.p. injection with insulin (0,75 U/kg bodyweight). Blood samples were taken by tail-cut at baseline and after 15, 30, 45, 60, 90 and 120 minutes of insulin administration. Blood glucose concentration were measured with a blood glucose meter (Accu-Chek Avia; Roche Diagnostics, Almere, The Netherlands).

### Statistical analysis

All data are represented as mean ± SD except for the arthritis data which, is represented as mean ± SEM. Data were analyzed using, where appropriate, the Student's t-test, ANOVA or 2-way ANOVA followed by the Tukey *post hoc* test (SPSS, Chicago, IL, USA). A p-value <0.05 was considered significant.

## Results

### The blood glucose kinetics test does not interfere with arthritis development

**Figure 1:**
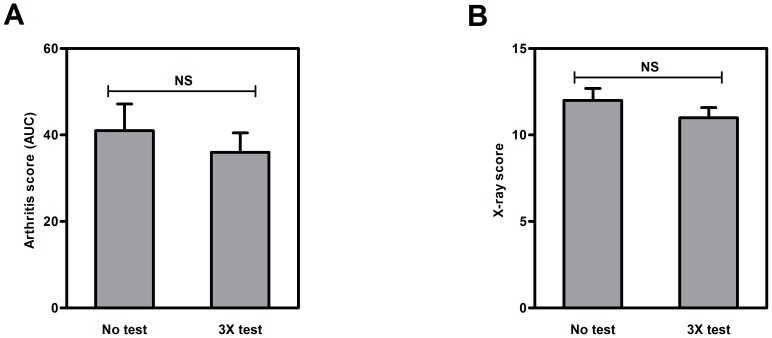
Arthritis development. Arthritis development in mice subjected to blood glucose kinetics three times and in mice in which no experiments were performed. (**A**) AUC of the overall arthritis score, corrected for base line, after 21 days. (**B**) X-ray analysis to assess bone destruction. Results are presented as mean ± SEM (n = 20). No significant differences between the two groups was observed. NS  =  not significant.

Before reliable statements could be made regarding prednisolone-induced effects on arthritis development and glucose kinetics in mice, we wanted to confirm that the blood glucose kinetics protocol itself did not influence arthritis development. Therefore, we first investigated if performing this protocol three times in arthritic mice would affect arthritis development. Figure 1 shows that a triple blood glucose kinetic test in three weeks time did not affect AUC of arthritis scores and X-ray scores in the CIA mouse model.

### Assessing the effects of both arthritis development and prednisolone treatment on glucose kinetics

**Figure 2:**
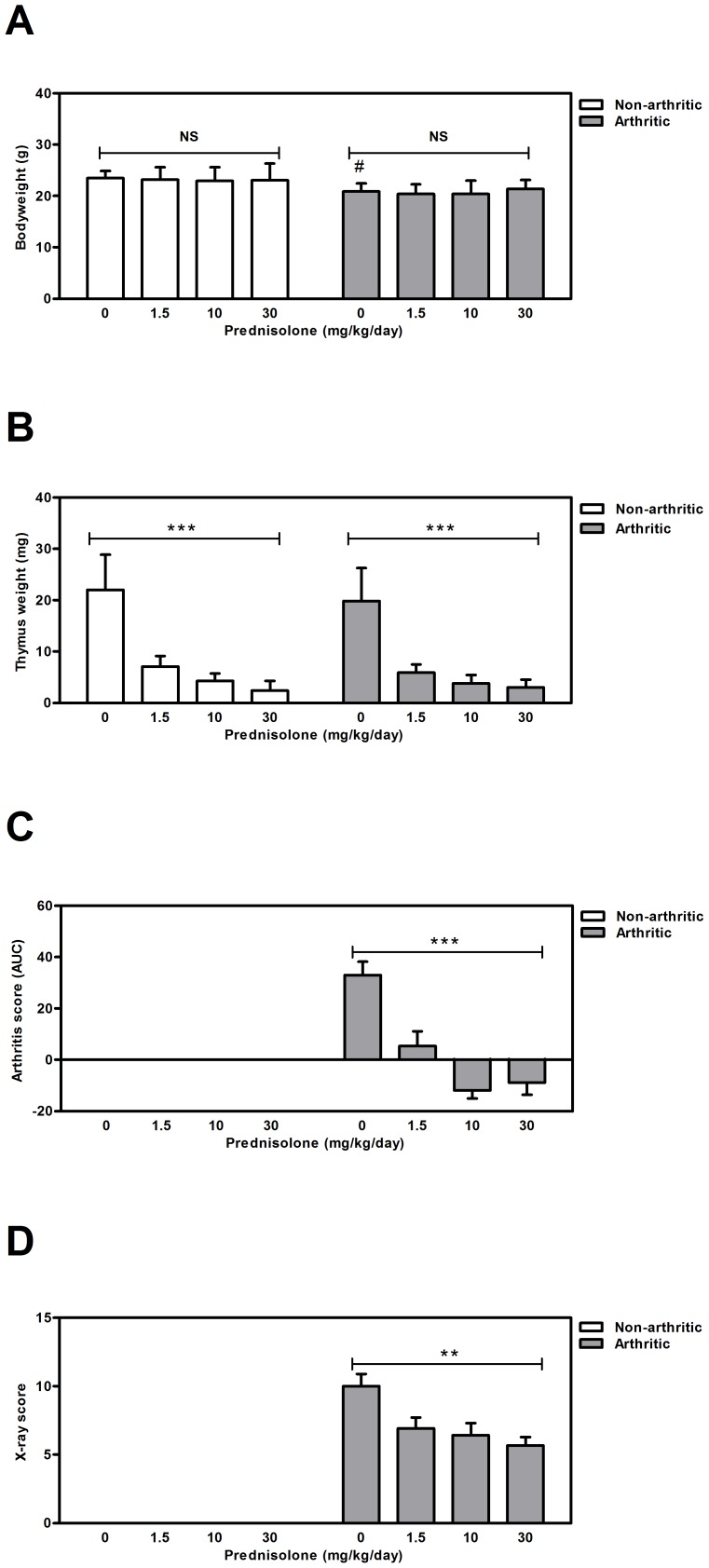
Effects of prednisolone on bodyweight, thymus weight and arthritis development after 21 days of treatment. (**A**) Bodyweight (**B**) thymus weight (**C**) AUC of the overall arthritis score (**D**) X-ray analysis to assess bone destruction. Results are represented as means ± SD for bodyweight and thymus weight and as mean ±SEM for AUC arthritis score and X-ray score and are outlined for each experimental group (n = 12). * Significant difference p≤0.05, ** significant difference p≤0.01, *** significant difference p≤0.001, NS  =  not significant.

Blood glucose kinetics was used to assess both arthritis-induced and prednisolone-induced metabolic dysfunction and was performed three times: at day 0 (before prednisolone treatment) and after 7 and 21 days of treatment. Before treatment, bodyweights were significantly lower in arthritic mice when compared to non-arthritic mice (p<0.0001) (Table 2). This decrease in bodyweight is caused by the active inflammatory state of these arthritic mice as was previously reported [24]. Even though arthritic mice have lower bodyweights compared to control mice, treatment with several doses of prednisolone for 21 days did not have any effect on bodyweight in both groups compared to vehicle treated mice (Table 2 and Fig. 2A).

**Table 2:** Treatment parameters for both non-arthritic and arthritic mice treated with prednisolone for 21 days.

	Non-arthritic mice				Arthritic mice			
Prednisolone (mg/kg/d)	0	1.5	10	30	0	1.5	10	30
Bodyweight (g) day 0	23.0±1.9	24.3±1.9	24.3±2.0	24.8±1.7	21.0±1.5#	20.7±2.5	20.5±1.5	20.3±1.6
Bodyweight (g) day 7	22.5±1.4	23.3±2.3	22.8±2.4	23.5±1.5	20.4±2.2#	20.1±2.1	20.7±1.8	20.2±1.6
Bodyweight (g) day 21	23.1±2.6	23.2±2.4	22.7±2.7	23.0±3.1	20.8±1.5#	20.1±2.1	20.4±2.6	21.6±1.8
Glucose (mM) day 0	5.2±1.4	4.9±1.5	5.9±1.9	6.3±2.5	5.0±1.1	4.4±1.1	5.1±1.2	4.7±2.0
Glucose (mM) day 7	6.5±1.6	5.1±1.2	4.6±1.1*	4.3±1.1*	5.4±1.0	4.7±1.0	4.7±0.9	4.3±1.7
Glucose (mM) day 21	6.7±1.2	5.9±1.0	5.6±1.2	4.9±0.7*	5.5±0.9	4.6±0.7*	4.7±1.0*	5.1±0.3
MCR (ml/kg/min) day 0	14.4±3.2	15.2±2.6	13.4±3.5	11.7±2.2	19.1±3.3#	19.2±3.2	19.0±1.8	17.9±3.4
MCR (ml/kg/min) day 7	15.9±2.2	15.3±2.8	16.1±2.6	17.4±1.5	17.2±2.4	18.4±3.0	17.6±1.5	16.8±2.9
MCR (ml/kg/min) day 21	11.5±1.6	12.1±1.6	11.5±1.5	12.6±1.2	14.4±1.4#	14.1±1.2	14.3±1.1	14.0±1.3
EGP (µmol/kg/min) day 0	71.2±10.9	72.5±22.1	75.3±24.1	72.0±27.7	94.3±24.7#	82.0±12.2	95.8±19.5	90.7±7.5
EGP (µmol/kg/min) day 7	100.9±18.6	78.8±23.1	73.0±15.6*	73.1±15.2*	92.0±14.7	84.7±19.2	80.8±9.7	81.3±14.2
EGP (µmol/kg/min) day 21	76.7±13.5	66.8±10.3	61.9±7.3*	58.5±10.2*	84.1±13.9	68.0±7.5*	65.8±10.1*	71.0±7.7*

Both non-arthritic and arthritic mice are treated with prednisolone (0, 1.5, 10 and 30 mg/kg/day) for 21 days. Each experimental group consists of 12 mice; in total 48 non-arthritic and 48 arthritic mice were included. Values represent means ± SD during the blood glucose kinetics test except BW, which is represented as means ± SD before the test.

* Significant difference (p≤0.05) when compared to the placebo-treated group of that same parameter.

# Significant difference (p≤0.05) when compared to the placebo-treated non-arthritic mice.

Prednisolone treatment induced apoptosis of thymocytes, thereby severely reducing thymus weight dose-dependently [25]. No differences in thymus weight were observed between arthritic and non-arthritic mice. Treatment with 1.5, 10 or 30 mg/kg prednisolone per day significantly reduced thymus weight in a dose-dependent manner respectively with 68%, 80% and 89% (p<0.0001) in the non-arthritic control group and with 70%, 81% and 85% (p<0.0001) in the arthritic group (Fig. 2B). These reductions are indicative for sufficient prednisolone exposure in these treatment groups. No differences in thymus weight reduction between arthritic and non-arthritic mice was observed.

To confirm the anti-arthritic properties of prednisolone, arthritic mice were monitored for arthritis score and X-ray score. Prednisolone dose-dependently reduced disease severity since a significant reduction of the AUC arthritis score covering the entire treatment period was observed (p<0.0001) (Fig. 2C). X-ray analysis of the arthritic joints indicate severe cartilage and bone destruction in the placebo-treated arthritic animals. In line with previous results [6], prednisolone significantly reduced the rate of cartilage and bone destruction in a dose-dependent manner (p = 0.0029) (Fig. 2D). As expected, the non-arthritic control mice that were mock immunized with saline and three weeks later mock boosted with saline, did not develop arthritis.

**Figure 3:**
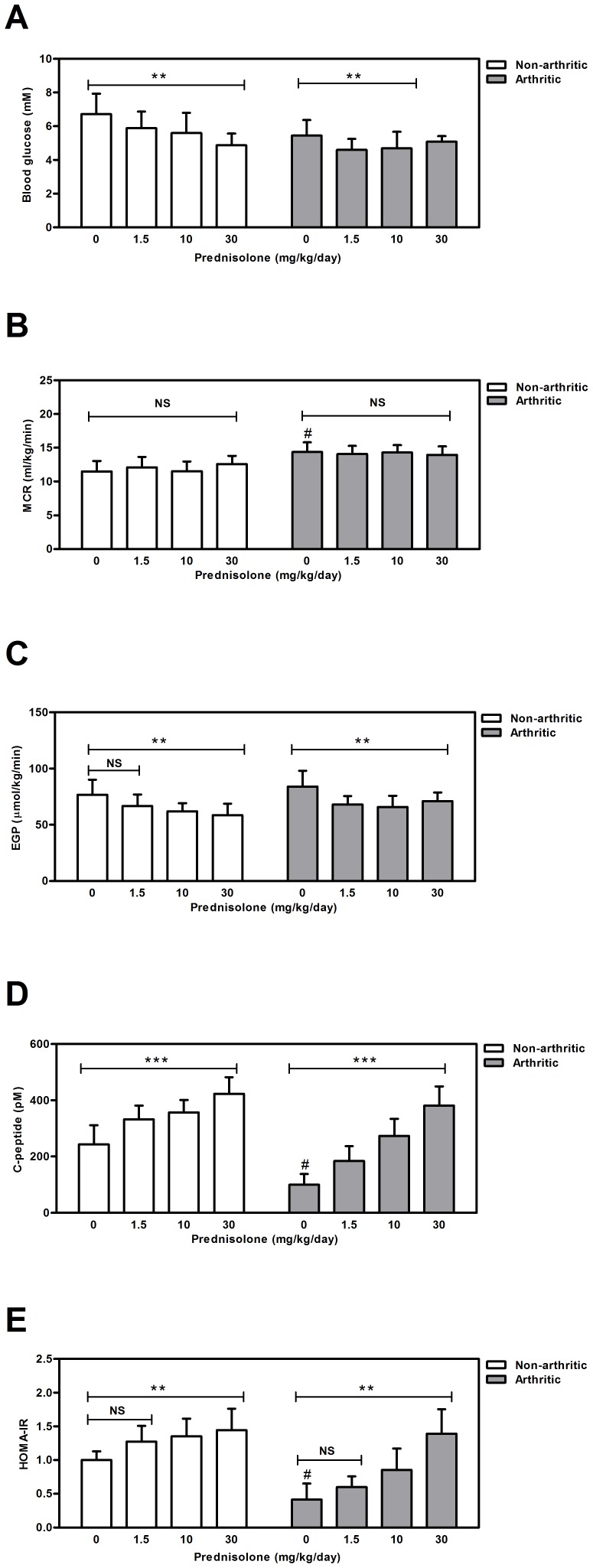
Effects of prednisolone on glucose kinetics after 21 days of treatment. (**A**) Blood glucose concentrations (**B**) MCR (**C**) EGP levels MCR (**D**) C-peptide concentrations (**E**) C-peptide HOMA-IR. Results represent means ± SD and are outlined for each experimental group (n = 12). * Significant difference p≤0.05, ** significant difference p≤0.01, *** significant difference p≤0.001, ^#^significant difference (p≤0.05) when compared to the placebo-treated non-arthritic mice, NS  =  not significant.

No differences were observed in blood glucose concentrations between arthritic and non-arthritic mice before treatment (Table 2), and after 21 days placebo treatment (p = 0.1). (Fig. 3A). A minimal but significant decrease in blood glucose concentrations was observed in non-arthritic mice after treatment with several doses of prednisolone for three weeks (p = 0.0064) (Fig. 3A). Also a slightly but significant decrease in blood glucose concentrations was observed in arthritic mice after treatment with several doses of prednisolone (p = 0.0012) (Fig. 3A).

The glucose kinetics parameters MCR (Metabolic Clearance Rate) and EGP (Endogenous Glucose Production) were calculated by modeling the wash-out of injected D-[6,6-^2^H_2_]-glucose. MCR was increased in arthritic mice when compared to non-arthritic before treatment started (p<0.0001) (Table 2). Prednisolone did not affect MCR in both non-arthritic or arthritic mice (Fig 3B). Interestingly, even though no effects were seen upon three weeks of prednisolone treatment between the groups, MCR is lowered in the arthritic group over time. For the non-arthritic group, MCR is increased at day 7 but decreased at day 21 when compared baseline (Table 2 and Fig. 3B)

Before treatment started the EGP in arthritic mice was significantly higher than in non-arthritic mice (p<0.0001), but decreased after 21 days of placebo treatment (Table 2), leveling the difference between placebo-treated arthritic and placebo-treated non-arthritic mice after 21 days of treatment (p = 0.2) (Table 2 and Fig. 3C). Prednisolone treatment for 21 days significantly decreased EGP in both non-arthritic (p = 0.006) and arthritic mice (p = 0.002) (Fig. 3C).

Surprisingly, after three weeks of treatment, plasma C-peptide concentrations of placebo treated arthritic mice were significant lower than those of placebo-treated non-arthritic mice (p = 0.0006). C-peptide concentrations were strongly increased in a dose-dependent manner by prednisolone in both non-arthritic (p<0.0001) and arthritic mice (p<0.0001) implying increased insulin production (Fig. 3D).

From the fasting blood glucose and C-peptide concentrations, the HOMA-IR was calculated to evaluate insulin resistance. Remarkably, the HOMA-IR showed that placebo-treated arthritic mice are 2.4 times more insulin sensitive than placebo-treated non-arthritic animals (p-value = 0.0007) (Fig. 3E). According to the HOMA-IR data, prednisolone treatment significantly reduced insulin sensitivity in a dose-dependent manner in both non-arthritic (p = 0.028) and arthritic mice (p = 0.0001 after correction for differences in arthritis scores between the groups). After treatment with prednisolone (30 mg/kg/d) for 21 days, both groups were equally sensitive to insulin (Fig. 3E).

### Validating the blood glucose kinetics results by using the insulin tolerance test

**Figure 4:**
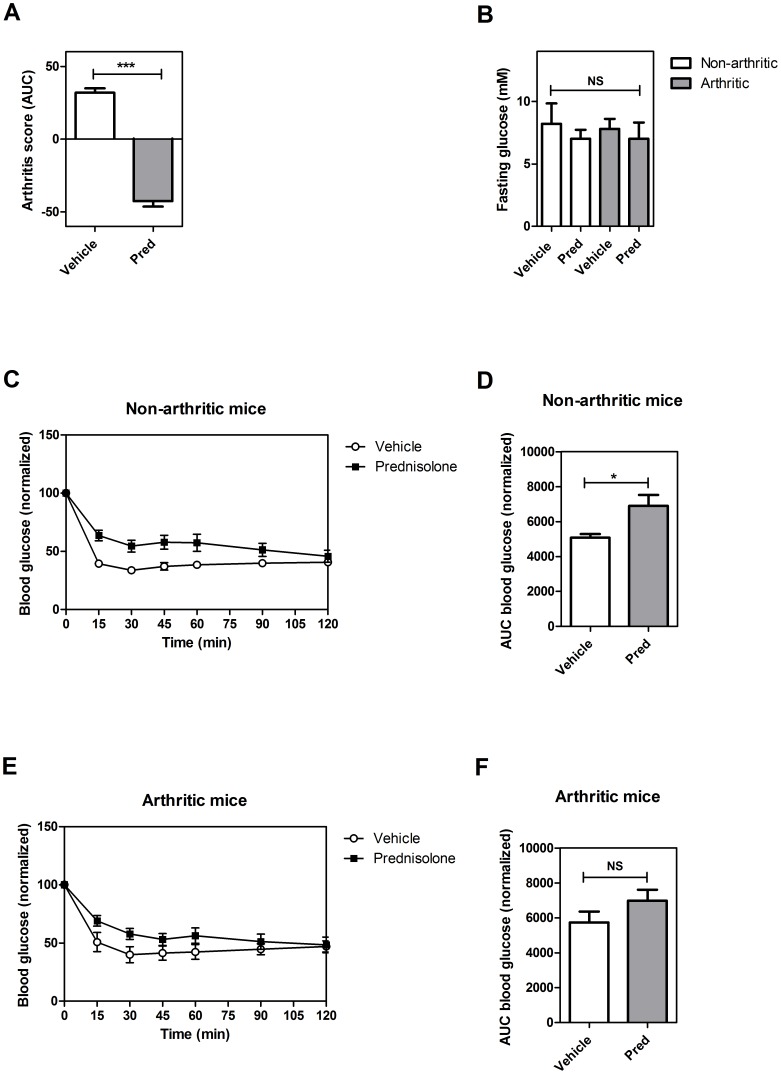
Assessment of insulin sensitivity by the use of the insulin tolerance test (ITT). (**A**) AUC of the overall arthritis score, corrected for base line, after 21 days. (**B**) Blood glucose concentrations of non-arthritic and arthritic mice treated with prednisolone or vehicle. (**C**) Blood glucose values during the ITT of non-arthritic mice treated with prednisolone or vehicle. (**D**) ITT AUC values of non-arthritic mice treated with prednisolone or vehicle. (**E**) Blood glucose values during the ITT of arthritic mice treated with prednisolone or vehicle. (**F**) ITT AUC values of arthritic mice treated with prednisolone or vehicle.). * Significant difference p≤0.05, *** significant difference p≤0.001, NS  =  not significant.

As described above, results from the blood glucose kinetic model showed that prednisolone treatment significantly reduced insulin sensitivity in both non-arthritic and arthritic mice. To validate these results, we repeated the experiment and assessed insulin resistance by the use of a insulin tolerance test (ITT). Both non-arthritic and arthritic mice were treated either with prednisolone (30 mg/kg/day) in vehicle or with vehicle alone for 21 days. After treatment, an ITT was performed. Prednisolone reduced disease severity as shown by a significant reduction of the AUC arthritis score (p<0.0001) (Fig. 4A). Fasting glucoses were not significant different between groups (Fig. 4B). Results from the ITT demonstrated that non-arthritic mice treated with vehicle were more insulin sensitive than non-arthritic mice treated with prednisolone (Fig. 4C). The area under the curve showed that differences between prednisolone- and vehicle treated animals was significant (p = 0.012) (Fig. 4D). In arthritic animals, a clear trend is visible suggesting that vehicle-treated mice are more insulin sensitive than prednisolone-treated mice (Fig. 4E). However, the AUC shows that differences are not significant (Fig. 4F). Taken together, data from the glucose kinetics test and the ITT showed that prednisolone treatment most likely reduces insulin sensitivity in both non-arthritic and arthritic mice.

### Assessing the effects of ORG 37663 treatment on glucose kinetics

To distinguish between the direct effect of prednisolone on metabolic processes versus indirect effects on metabolism via the suppressed inflammatory condition by prednisolone, we performed an experiment in which arthritic and non-arthritic mice were treated with prednisolone or ORG 37663. The latter compound is a steroid that was previously shown to have anti-inflammatory properties and effectively reduces arthritis in the CIA model. ORG 37663 mediates its effects through a mechanism that is independent of GR binding [6]. Therefore, ORG 37663 was used in a head-to-head comparison to prednisolone in one experiment to study to what extent effects of prednisolone on glucose kinetics are due to its anti-inflammatory properties or due to a direct effect through GR on metabolic pathways.

**Figure 5:**
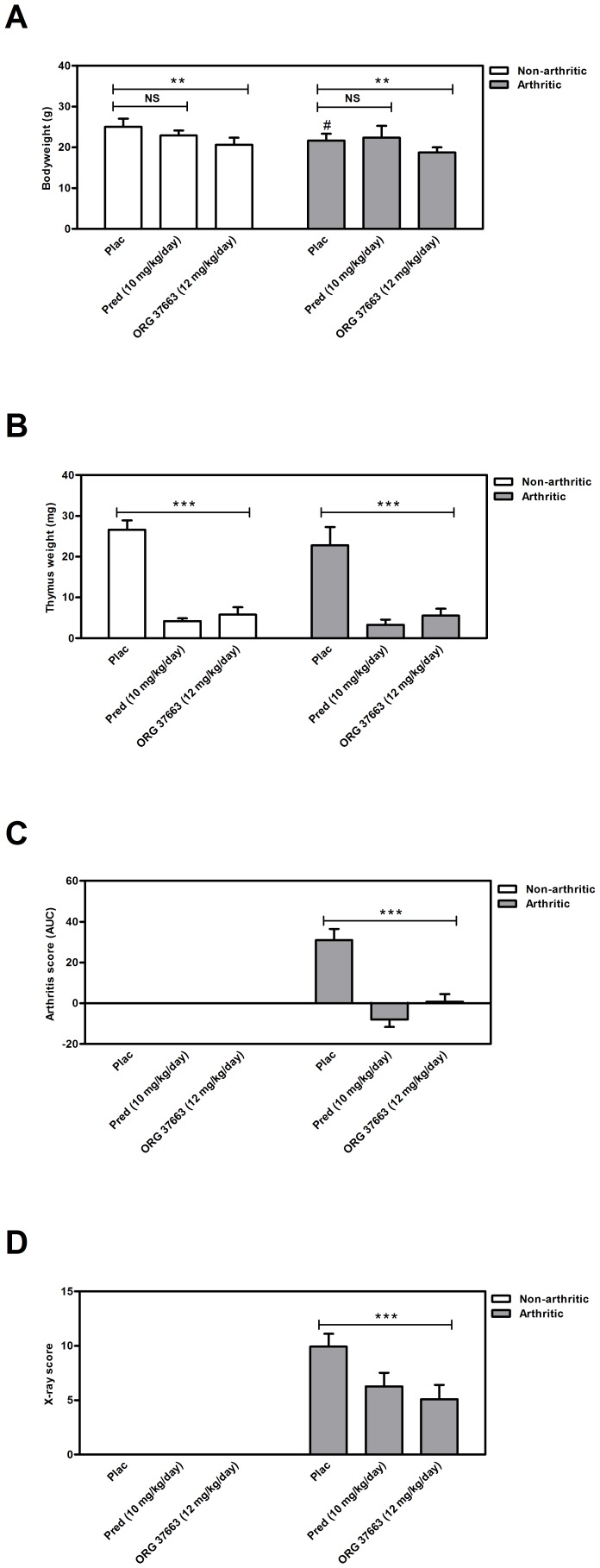
Effects of ORG 37663 and prednisolone on bodyweight, thymus weight and arthritis development after 21 days of treatment. (**A**) Bodyweight (**B**) thymus weight (**C**) AUC of the overall arthritis score (**D**) X-ray analysis to assess bone destruction. Results are represented as means ± SD for bodyweight and thymus weight and as mean ±SEM for AUC arthritis score and X-ray score and are outlined for each experimental group (n = 12). * Significant difference p≤0.05, ** significant difference p≤0.01, *** significant difference p≤0.001, ^#^significant difference (p≤0.05) when compared to the placebo-treated non-arthritic mice, NS  =  not significant.

Similar to the previous experiment, bodyweights before treatment were significantly lower in arthritic mice compared to non-arthritic mice (p = 0.0001) (Table 3). Non-arthritic mice were found to have significant lower bodyweights after treatment with ORG 37663 but not after treatment with prednisolone (Fig. 5A).

**Table 3:** Treatment parameters for both non-arthritic and arthritic mice treated with placebo, prednisolone (10 mg/kg) or ORG 37663 (12 mg/kg) for 21 days.

	Non-arthritic mice			Arthritic mice		
	Placebo	Prednisolone	ORG 37663	Placebo	Prednisolone	ORG 37663
Bodyweight (g) day 0	23.5±1.5	23.2±1.0	25.0±2.4	20.9±1.3#	21.5±2.9	20.8±1.3
Bodyweight (g) day 7	24.3±1.6	22.6±1.2	23.7±1.6	21.4±2.2#	21.4±2.7	21.7±1.9
Bodyweight (g) day 21	25.1±1.9	22.9±1.2	20.5±1.6*	21.6±1.7#	22.4±2.9	18.7±1.2*
Glucose (mM) day 0	3.5±1.1	4.6±1.7	3.6±1.8	4.8±1.0	5.0±1.1	4.5±0.9
Glucose (mM) day 7	5.3±1.9	4.4±1.2	5.2±1.4	5.7±0.7	4.6±1.0	6.0±0.8
Glucose (mM) day 21	5.2±1.4	4.9±0.9	4.6±1.5	5.9±1.3	5.2±0.8	4.7±0.7*
MCR (ml/kg/min) day 0	17.8±9.4	18.6±8.3	15.6±10.2	19.0±4.1	18.9±2.8	13.4±10
MCR (ml/kg/min) day 7	11.6±5.6	14.1±4.9	9.8±6.3	17.0±1.9#	16.7±2.6	8.8±6.9*
MCR (ml/kg/min) day 21	16.5±1.8	12.0±1.1*	13.2±1.9*	12.3±1.4#	12.9±1.8	13.6±2.4
EGP (µmol/kg/min) day 0	71.1±13.3	84.6±16.1	68.5±19.5	92.3±25.5#	95.1±21.5	89.9±15.9
EGP (µmol/kg/min) day 7	72.7±25.6	65.5±13.2	65.9±9.8	94.1±14.3#	74.0±7.7*	78.7±12.2*
EGP (µmol/kg/min) day 21	84.4±19.4	58.4±8.3*	58.6±12.9*	73.7±19.4	66.0±10.8	63.6±16.4

Both non-arthritic and arthritic mice are treated with placebo, prednisolone (10 mg/kg/day) or ORG 37663 (12 mg/kg/day) for 21 days. Each experimental group consists of 12 mice; in total 36 non-arthritic and 36 arthritic mice were included. Values represent means ± SD during the blood glucose kinetics test except BW, which is represented as means ± SD before the test.

* Significant difference (p≤0.05) when compared to the placebo-treated group of that same parameter.

# Significant difference (p≤0.05) when compared to the placebo-treated non-arthritic mice.

In the non-arthritic control group, treatment with 10 mg/kg prednisolone or 12 mg/kg ORG 37663 per day reduced thymus weight respectively with 84% (P<0.0001) and 78% (P<0.0001) (Fig. 5B). In the arthritic group thymus weight was reduced with 86% (P<0.0001) and 76% (P<0.0001), again indicative for sufficient compound exposure. In arthritic mice, both arthritic score and X-ray score were significantly decreased (P<0.0001 and p = 0.0279 respectively) after treatment with either prednisolone or ORG 37663 (Fig. 5C-D).

**Figure 6:**
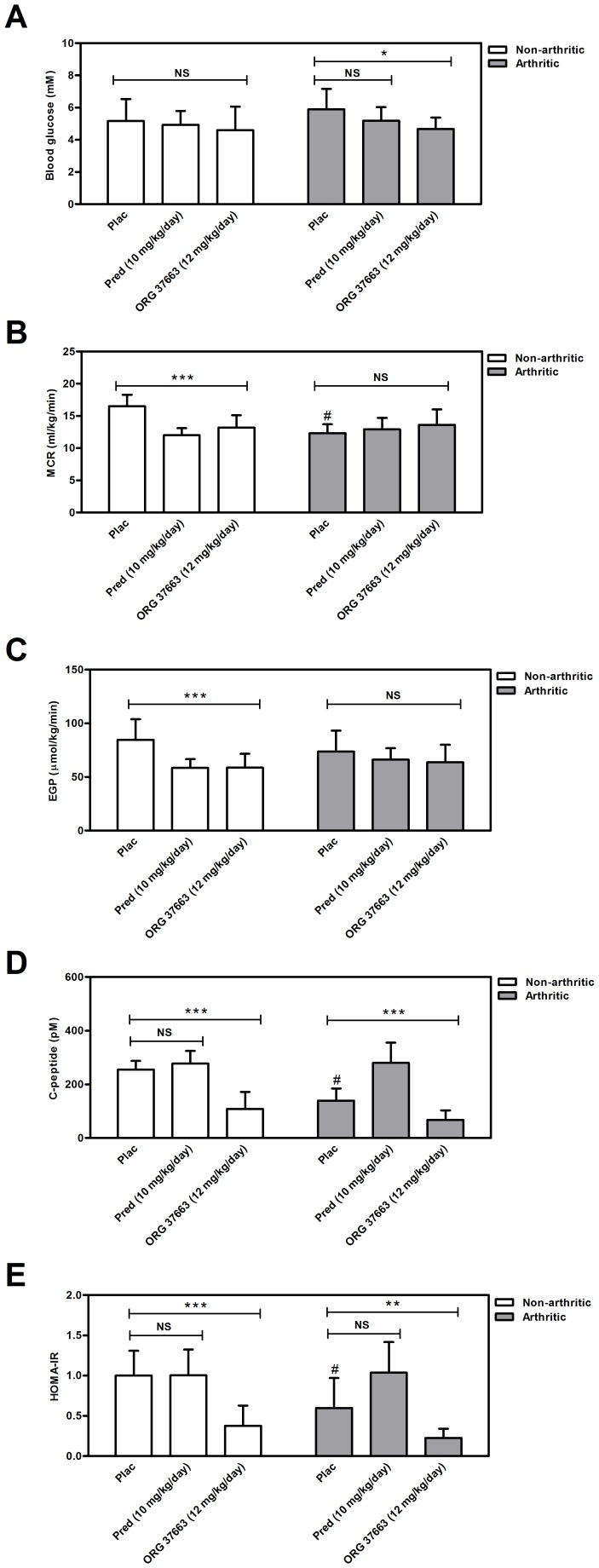
Effects of ORG 37663 and prednisolone on glucose kinetics after 21 days of treatment. (**A**) Blood glucose concentrations (**B**) MCR (**C**) EGP levels (**D**) C-peptide concentrations (**E**) C-peptide HOMA-IR. Results represent means ± SD and are outlined for each experimental group (n = 12). * Significant difference p≤0.05, ** significant difference p≤0.01, *** significant difference p≤0.001, ^#^significant difference (p≤0.05) when compared to the placebo-treated non-arthritic mice, NS  =  not significant.

Arthritic mice showed slight but significant lower blood glucose concentrations after ORG 37663 treatment in comparison to placebo treatment (p = 0.036). In both the non-arthritic and arthritic groups of mice, no effect of prednisolone on blood glucose concentrations was detected (Fig. 6A). Non-arthritic mice treated with prednisolone or ORG 37663 both had a significant lower MCR compared to placebo treated mice (p<0.0001), while this effect was not observed in the arthritic mice after treatment (Fig. 6B). This is probably caused by the fact that MCR was already decreased in arthritic mice after 21 days of treatment with placebo compared to non-arthritic mice treated with placebo (p<0.0001). The same effect was observed for EGP: in non-arthritic mice the EGP levels decreased by prednisolone (p = 0.0006) or ORG 37663 treatment (p = 0.036) (Fig.6C) and a same trend was visible in arthritic mice, but due to the fact that the placebo-treated arthritic mice already showed some neutralization of the increased EGP levels, the effects of prednisolone and ORG 37663 on EGP in these mice was less obvious (as was seen in the previous experiment).

As was observed in the prednisolone dose-response experiment, C-peptide concentrations from arthritic mice treated with placebo were significant lower compared to the non-arthritic mice treated with placebo after three weeks of treatment (p-value = 0.0047) (Fig. 6D). The ORG 37663 compound caused a decrease in C-peptide concentrations in both non-arthritic (p<0.0001) and arthritic mice (p<0.0001) when compared to prednisolone (Fig. 6D). As a consequence of the low C-peptide concentrations after treatment with ORG 37663, both arthritic (p = 0.0022) and non-arthritic mice (p = 0.0002) showed a tremendous increase in insulin sensitivity as calculated by the HOMA-IR (Fig. 6E). These data indicate that treatment with ORG 37663 improved insulin sensitivity in mice compared to prednisolone-treated mice.

## Discussion

There is an urgent need for improved GCs that are as effective as classical GCs, but have a better safety profile. However, a chronic *in vivo* inflammatory model in which (experimental) GCs can be studied for both their effectiveness and the induction of metabolic side effects is currently unavailable. Goal of the present study was to develop such an *in vivo* inflammatory model for efficacy and metabolic adverse effects at the same time. Therefore we have integrated a single-pool, first order kinetic protocol to examine blood glucose kinetics, in the well-established collagen-induced arthritis (CIA) mice model, a chronic inflammatory model which is often used to monitor efficacy of experimental anti-inflammatory compounds. We were able to measure effects on glucose kinetics without influence on arthritis development. Importantly, this indicates that we have successfully performed blood glucose kinetics in the CIA model enabling the quantification of both efficacy and effects on glucose metabolism in a single animal.

The ‘gold standard’ to investigate and quantify insulin resistance is the hyperinsulinemic euglycemic clamp (HIEC) [26]. However, seen from the perspective of laboratory animal welfare, performing such clamp studies within an inflammatory mouse model such as the CIA model is impossible. Measuring glucose kinetics by modeling the wash-out of *ip* injected D-[6,6-^2^H_2_]-glucose is performed under fasted conditions whereas the HIEC utilizes steady-state insulin concentrations that are supraphysiological [13], [27], [28]. Thus, the wash-out of ip-injected glucose presumably reflects a more physiological relevant situation. In addition, unlike the HIEC the blood glucose kinetics test can be applied repeatedly and is therefore applicable in longitudinal studies, such as the CIA model described here.

This study showed that prednisolone decreased disease severity and joint destruction in arthritic mice, thereby confirming the anti-inflammatory properties of this compound. Regarding the induction of metabolic effects, prednisolone slightly decreased fasted glucose concentrations and EGP, but strongly increased insulin production (as measured by the increase in C-peptide concentrations) at the same time in both non-arthritic and arthritic mice. This resulted in an increased HOMA-IR after prednisolone treatment, indicating the induction of insulin resistance. To validate these results, we repeated the experiment and measured insulin sensitivity by the use of an ITT. Results from the ITT showed that prednisolone induced insulin resistance in both non-arthritic and arthritic mice, thereby confirming our previous results obtained by the blood glucose kinetic model. Although the ITT is a more classical model to assess insulin resistance, it is only capable of measuring glucose metabolism in peripheral tissues. The additive value of the applied blood glucose kinetic model, *i.e*., the injection of D-[6,6-^2^H_2_]-glucose, is that this allowed for discrimination of the changes in glucose metabolism in peripheral tissues and the liver since the MCR reflects peripheral glucose metabolism and EGP reflects hepatic glucose production in fasted conditions. To our knowledge, no animal model was previously described which enables quantification of both anti-arthritic efficacy and longitudinal tissue-specific metabolic adverse effects in one animal in one chronic treatment protocol. In the field of GCs and the search for compounds with improved efficacy/safety profiles (SGRMs), such a model is of great value since it closely mimics the clinical RA practice. Interestingly, our results show that the overall effects of prednisolone on glucose kinetics were similar in both arthritic and non-arthritic animals indicating that the disease state in this model did not influence regulation of glucose homeostasis.

One reason to include both non-arthritic and arthritic mice in this study was to be able to distinguish between inflammation-induced and GC-induced metabolic dysfunction. Our results showed that arthritic mice produce less insulin than non-arthritic mice after treatment with placebo. It is known that arthritic mice have elevated circulating levels of the pro-inflammatory cytokines TNFα and IL-1β [14]. Proinflammatory cytokines have been shown to induce beta cell dysfunction [29]. In addition, chronic inflammation may reduce beta cell mass by stimulating apoptosis of beta cells [30]. Also factors related to the disease status may play a role in the difference in C-peptide production between arthritic and non-arthritic mice. For example, mice suffering from arthritis eat less and have to be more efficient with energy which might affect glucose metabolism. Nonetheless, based upon the fact that glucose concentrations did not change, the untreated arthritic mice in our study were still able to compensate for the inflammation-induced reduction of insulin secretion. Both severely arthritic mice and mice treated with a high dose of prednisolone are able to compensate for reduced insulin secretion and reduced insulin sensitivity respectively by keeping the MCR steady and even reduce EGP, which indicates a highly adaptive compensatory mechanism in both groups of mice.

Regarding GC-induced insulin resistance in mice, many conflicting results have been reported [27], [31]–[38]. Also the results described in this paper show discrepancies in results when compared to previously reported studies. For instance, we report a minimal decrease in glucose levels after treatment with prednisolone whereas other studies reported an increase of glucose levels after treatment with glucocorticoids [27], [31]–[34]. One reason explaining (at least partly) these differences in results is that different strains of mice were used. We used DBA1/J mice since this strain showed to be highly susceptible for inducing collagen-induced arthritis [39]. However, most studies use C57BL/6 mice to investigate GC-induced insulin resistance [27], [36]–[38], [40] and DBA1/J mice are hardly ever used in diabetic or metabolic model systems. Other reasons for differences in results between studies might be explained by differences in study design (e.g. fasting conditions), administered dose, treatment duration and/or route of administration.

In humans, GCs are well known to negatively affect glucose metabolism. Short-term clinical trials in healthy individuals have shown that GCs reduce hepatic and peripheral insulin sensitivity [5] and impair beta cell function [41]. Population-based studies showed that GC use was associated, in a cumulative dose-dependent manner, with the incidence of diabetes [42]. However, studies investigating the metabolic effects of GCs in RA patients yielded conflicting results. On the one hand, in cross-sectional studies in RA patients showed that GC exposure was related to impaired fasting insulin sensitivity [43] and tended to predict development of type 2 diabetes [44], [45]. On the other hand, the use of GCs by patients with chronic inflammatory conditions may improve glucose tolerance via anti-inflammatory effects, as has been demonstrated in a number of studies [46], [47]. Our results regarding glucose and C-peptide levels are in line with the results reported by den Uyl and co-workers. These authors investigated the dose-related effects of short-term GC treatment on glucose tolerance, beta cell function and insulin sensitivity in patients with early active RA. They showed that fasting C-peptide levels increased significantly after treatment with prednisolone while fasting glucose levels did not change [48]. These results regarding C-peptide and glucose levels are also observed in our experiments, indicating reduced insulin sensitivity. However, both the non-arthritic and arthritic mice were, after 21 days of prednisolone treatment, still able to compensate for their prednisolone-induced reduction in insulin sensitivity, resulting ultimately in no change in blood glucose concentrations. Most likely, the treatment period (3 weeks) in this combined model is too short to study a next stage in the development of diabetes, *i.e.*, hyperglycemia and beta-cell dysfunction. These results, together with the results reported by den Uyl and coworkers point to a delicate balance between the anti-inflammatory and diabetogenic effects in which dose and duration of GC treatment are crucial. Further studies will be needed to challenge this.

To be able to study to what extent the effects of prednisolone on glucose kinetics are due to its anti-inflammatory properties or due to a direct effect on glucose kinetics, mice were also treated with ORG 37663, a non-GC anti-inflammatory compound, and directly compared to prednisolone. This compound was chosen because of its strong anti-inflammatory effects that are not mediated through the GR. Although it is reported that this compound lacks anti-rheumatic activity in humans [49], Dulos and co-workers showed that treatment with ORG 37663 suppressed clinical arthritis score, reduced inflammatory infiltrates and decreased pro-inflammatory cytokines in collagen-induced arthritis mice [6]. Also our own data showed that the clinical arthritis score was strongly decreased after treatment with this compound. Therefore, we believe that this compound is suitable to compare GR- and non-GR-mediated effects in this in-vivo model. Treatment with ORG 37663 resulted in lower C-peptide concentrations when compared to prednisolone treatment in both non-arthritic and arthritic mice. This indicates that prednisolone mediates its effects on insulin production directly and not via indirect anti-inflammatory effects. However, the effects on glucose kinetics (MCR) and metabolism (EGP) seem to be due to the anti-inflammatory activity of prednisolone as treatment with another (non-GR agonistic) anti-inflammaroy drug (ORG 37663) showed the same effects. However, ORG 37663 also influenced glucose kinetics in non-arthritic mice, decreasing C-peptide levels. This indicate that the observed effects of ORG 37663 are not only mediated indirectly by suppressing inflammation but might also by directly influencing glucose kinetic pathways.

To conclude, we successfully integrated the whole body glucose test in the CIA mice model, which enabled us to measure both efficacy and metabolic (adverse) effects of (experimental) GCs in a chronic *in vivo* inflammatory setting. Thereby, it may prove a useful translational model for compound-induced metabolic derangements in patients suffering from RA or other (auto)immune diseases. This study showed that both inflammation and glucocorticoids affect insulin secretion and sensitivity but this did not lead immediately to the development of hyperglycemia and type 2 Diabetes Mellitus. Data from this study emphasizes the highly adaptive character of these mice and the complex network of several integrated mechanisms underlying the development of GC-induced and/or inflammation-induced metabolic dysfunction.
